# αT-catenin in restricted brain cell types and its potential connection to autism

**DOI:** 10.1186/s40303-016-0017-9

**Published:** 2016-06-21

**Authors:** Stephen Sai Folmsbee, Douglas R. Wilcox, Koen Tyberghein, Pieter De Bleser, Warren G. Tourtellotte, Jolanda van Hengel, Frans van Roy, Cara J. Gottardi

**Affiliations:** Department of Medicine, Northwestern University Feinberg School of Medicine, Chicago, IL 60611 USA; Department of Pediatrics, Northwestern University Feinberg School of Medicine, Chicago, IL 60611 USA; Department of Microbiology-Immunology, Northwestern University Feinberg School of Medicine, Chicago, IL 60611 USA; Department of Pathology, Northwestern University Feinberg School of Medicine, Chicago, IL 60611 USA; Department of Neurology, Northwestern University Feinberg School of Medicine, Chicago, IL 60611 USA; Department of Cellular and Molecular Biology, Northwestern University Feinberg School of Medicine, Chicago, IL 60611 USA; The Driskill Graduate Training Program in Life Sciences, Northwestern University Feinberg School of Medicine, 240 East Huron St., McGaw Pavilion, M-323, Chicago, IL 60611 USA; Department of Biomedical Molecular Biology, Molecular Cell Biology Unit, Ghent University, Ghent, Belgium; Inflammation Research Center, Flanders Institute for Biotechnology (VIB), B-9052 Ghent, Belgium; Department of Basic Medical Sciences, Faculty of Medicine and Health Sciences, Ghent University, Ghent, Belgium

**Keywords:** Alpha-T-catenin, Adherens junction, Autism, Alzheimer’s disease, Cerebellum, Choroid plexus, Ependyma, Schizophrenia

## Abstract

**Background:**

Recent genetic association studies have linked the cadherin-based adherens junction protein alpha-T-catenin (αT-cat, *CTNNA3*) with the development of autism. Where αT-cat is expressed in the brain, and how its loss could contribute to this disorder, are entirely unknown.

**Methods:**

We used the αT-cat knockout mouse to examine the localization of αT-cat in the brain, and we used histology and immunofluorescence analysis to examine the neurobiological consequences of its loss.

**Results:**

We found that αT-cat comprises the ependymal cell junctions of the ventricles of the brain, and its loss led to compensatory upregulation of αE-cat expression. Notably, αT-cat was not detected within the choroid plexus, which relies on cell junction components common to typical epithelial cells. While αT-cat was not detected in neurons of the cerebral cortex, it was abundantly detected within neuronal structures of the molecular layer of the cerebellum. Although αT-cat loss led to no overt differences in cerebral or cerebellar structure, RNA-sequencing analysis from wild type versus knockout cerebella identified a number of disease-relevant signaling pathways associated with αT-cat loss, such as GABA-A receptor activation.

**Conclusions:**

These findings raise the possibility that the genetic associations between αT-cat and autism may be due to ependymal and cerebellar defects, and highlight the potential importance of a seemingly redundant adherens junction component to a neurological disorder.

**Electronic supplementary material:**

The online version of this article (doi:10.1186/s40303-016-0017-9) contains supplementary material, which is available to authorized users.

## Background

The pathogenesis of autism spectrum disorder (ASD) is complex, likely reflecting interplay between an underlying genetic predisposition and environmental influence. Through recent advances in clinical genetic analysis of those with ASD, many novel genes have been identified as potentially important mediators of the disorder. One such gene, αT-catenin (αT-cat, *CTNNA3*), has been implicated to be associated with ASD by a large number of independent genetic analyses [1–9]. Specifically, several genome-wide association studies have linked single nucleotide polymorphisms of αT-cat/*CTNNA3* to ASD [1–3]. Moreover, studies of copy number variants demonstrated that those with ASD were more likely to have lost the αT-cat/*CTNNA3* gene [4–7]. Additionally, αT-cat has been linked to other neurologic disorders with autistic-like behaviors [8], and a recent familial study found that compound heterozygote truncating mutations in αT-cat protein were associated with ASD [9]. Despite these connections to ASD, the localization and roles of αT-cat in the brain remain unknown.

α-catenins are essential F-actin-binding proteins of the cadherin/catenin adhesion complex, the major cell-cell adhesion system in tissues throughout the body [10]. αE-catenin (αE-cat, *CTNNA1*) is the most well studied member of this family, as it is nearly universally expressed [11]. Mouse knockout studies establish its requirement for organ structure and function across various tissue types [12–15]. αT-cat is the most tissue-restricted and developmentally dispensable member of the family, as αT-cat knockout mice are viable and fertile [16], with expression apparently restricted primarily to the heart and testis [11]. Interestingly, there is some evidence that αT-cat may impact other neurologic diseases, including Alzheimer’s disease (AD) [17–19] and schizophrenia related to maternal cytomegalovirus (CMV)-infection [20, 21]. But despite all these genetic associations, the majority of research on αT-cat is limited to its function in cardiomyocytes [16, 22–24] where it is abundantly expressed. While immunoblot detection of αT-cat has been observed in the brain [9], the primary cell type and plausible contribution to these neurological diseases have remained unexplored.

## Methods

### Mice

All experiments using animals were approved by the Northwestern University IACUC, and the care of experimental animals was in accordance with institutional guidelines. αT-cat KO C57BL/6 mice (obtained via Dr. Glenn Radice, Thomas Jefferson University, Philadelphia, PA) [16] were bred with C57BL/6 WT to create heterozygote, C57BL/6 breeders. WT and αT-cat KO mice used for experimentation were littermates or descendants from littermates. They were genotyped using αT-cat-KO specific primers, which generate separate bands in KO and WT mice via PCR: F: 5′- TCTATTTTTGAGGCTGTCG-3′; R: 5′-CAAACTTATGCGTGGTG-3′. αT-cat KO was confirmed by PCR, distinguishing from heterozygotes, showing absence of a band generated by WT-specific primers: F: 5′-CCACCCCTGATATGACCTGTAG-3′; R: 5′-TCCCCAGGAATCAAGTCGTT-3′.

### Histology and immunofluorescence

Mice were anesthetized and subjected to intracardiac perfusion with saline, followed by perfusion of 4 % paraformaldehyde fixative. Whole brains were removed and post-fixed in 4 % formaldehyde and embedded in paraffin blocks. For immunofluorescence, tissue sections were deparaffinized and antigens were retrieved by boiling in citrate buffer for 30 min. Fluorescent images were captured using a Zeiss Axioplan epifluorescence microscope. Primary antibodies used were: BD Biosciences mouse anti-α-E-catenin (#610193), rabbit anti-α-T-catenin (polyclonal #952) [11], rat anti-α-T-catenin (monoclonal, 1159_12A4S4), Millipore mouse anti-α-T-catenin (MAB2087), BD Transduction Laboratories mouse anti-E-cadherin (610182), BD Transduction Laboratories mouse anti-N-cadherin (#610920), Santa Cruz Biotechnology rabbit anti-β-catenin (H-102, SC-7199), Millipore rabbit anti-Connexin-43 (AB1728), Santa Cruz Biotechnology rabbit anti-p120-catenin (S-19, SC-1101), Santa Cruz Biotechnology goat anti-δ-catenin (C-20, SC-16512), Invitrogen mouse anti-ZO-1 (33-9100), Synaptic Systems rabbit anti GABRA2 (224-103), Santa Cruz Biotechnology goat anti-doublecortin (C-18, SC-8066), Dako rabbit anti-GFAP (Z0334), Leica Biosystems rabbit anti-Ki-67 (NCL-Ki67p), Sigma mouse anti-Tuj1 (T8660), Swant rabbit anti-Calbindin D28K (CB-38). Secondary antibodies used were: Alexa Fluor 488/568 goat anti-mouse/rabbit (Life Technologies) and Alexa Fluor 568 donkey anti-goat (Life Technologies). Tissue processing was supported by the Northwestern University Mouse Histology and Phenotyping Laboratory (MHPL) and a Cancer Center Support Grant (NCI CA060553). H&E histology was performed by the MHPL.

### Western blotting

Whole brains, from mice 7-8 weeks old, were lysed in T-PER Tissue Protein Extraction Reagent (ThermoFisher) with Protease and Phosphatase Inhibitor Mini Tablets (Pierce). Tissue was homogenized with tissue grinder pestle. Cerebral cortex and cerebellum tissue was carefully dissected to ensure no contamination from ventricular structures. Samples were run by SDS-PAGE, then transferred to nitrocellulose membrane. For western blot, primary antibodies used were: BD Biosciences mouse anti-α-E-catenin (#610193), rabbit anti-α-T-catenin (polyclonal, #952), BD Biosciences mouse anti-N-cadherin (#610920), Santa Cruz Biotechnology rabbit anti-β-catenin (H-102, SC-7199), Santa Cruz Biotechnology mouse anti-δ-catenin (40.1, SC-81793), Santa Cruz Biotechnology goat anti-doublecortin (C-18, SC-8066), Cell Signaling rabbit anti-α-N-catenin (2163S), Alomone rabbit anti-GABRA2 (AGA002), and Sigma mouse anti-beta-tubulin (T4026). The secondary antibodies were: LI-COR IRDye680 Donkey anti-rabbit (926-68073) and IRDye800 Donkey anti-mouse (926-32212). Imaging performed using the Odyssey Infrared Imaging System (LI-COR).

### Human tissue

No consent was necessary for the human data, as de-identified human brain tissue was obtained during autopsy, with an exemption granted from the Northwestern IRB. For both the ependymal and cerebellar tissue, there was no evidence of pathologic damage to the region. Tissues were processed using the Robert H. Lurie Comprehensive Cancer Center of Northwestern University Pathology Core Facility.

### Immunofluorescence quantification

After staining for α-E-catenin as above, the total fluorescent intensity density of exposure-matched images was measured. This was done by outlining the entirely of the ependymal cell layer along the visible cellular borders of the anterior lateral ventricles using ImageJ, and measuring the density of the signal averaged across this area. Although the majority of the α-E-catenin signal was present near the apical junctions of the cells, the entire ependymal layer was quantified to reduce any potential bias in quantification. Two measurements were performed per mouse, one for each ventricle in the section of each coronally sectioned brain tissue. Therefore, for each group, 6 measurements were performed, with 3 mice per group. For the quantification of Ki-67 positive cells, the total numbers of Ki-67 cells were counted from each ventricular area from coronal H&E-stained sections of the anterior lateral ventricles. Both ventricles present were quantified, and the mean of both Ki-67 positive counts was utilized per mouse for quantification.

### Ventricle size quantification

**Fig. 1:**
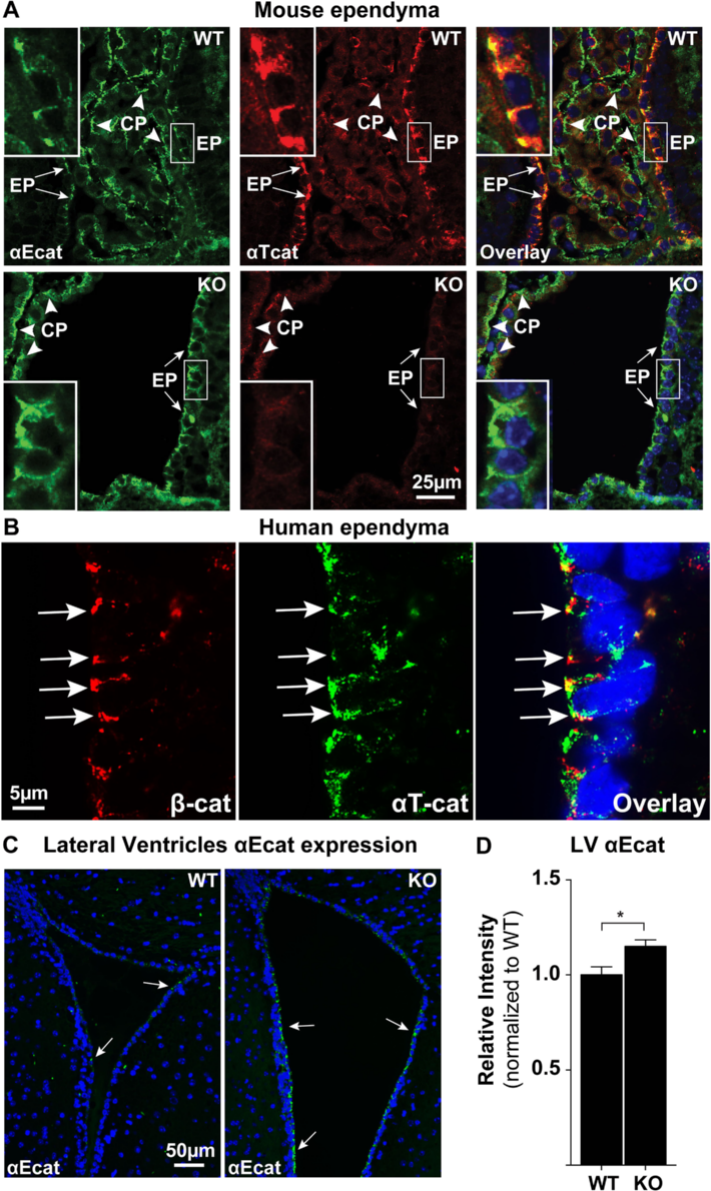
αT-cat is localized to ependymal, but not choroid plexus, epithelial cell junctions. **a** Double immunofluorescence labeling of the perihippocampal third ventricle of WT and αT-cat KO mice with antibodies to either αT-cat or αE-cat. αE-cat is found in both ependymal (EP) cell-cell junctions (arrows, enlarged in white boxes) and choroid plexus (CP) (*arrowheads*), whereas αT-cat is detected in only ependymal cells. **b** Immunofluorescence of the lateral ventricle proximal to the subventricular zone of an adult human. β-catenin is found at ependymal cell-cell junctions with αT-cat (*arrows*). **c** Immunofluorescence staining of αE-cat in the anterior lateral ventricles of WT and αT-cat KO mice, quantified in (**d**), *n* = 6. Hoechst-stained nuclei in blue. *p < 0.05, Student’s *t*-test. Error bars = S.E.M

For the quantification of the size of the lateral ventricles, the ventricular lumen area from coronal H&E-stained sections was quantified using ImageJ. Mice were age-matched, sex-matched, and were the progeny of littermates. Only intact lateral ventricles, clearly adjacent to the hippocampus (as in Fig. 1) were measured. Because the lateral ventricles from other sections of the brain, particularly in the anterior lateral ventricles, showed substantial variability with few discrete histological landmarks, the perihippocampal lateral ventricles were chosen for quantification to minimize potential bias and increase the consistency of the measurements. If more than one ventricle was intact and visible in the tissue section, the mean of both ventricles was utilized per mouse for quantification.

### RNA isolation

After dissection of the brain, cerebellar tissue (*n* = 3) was used for RNA extraction using TRIzol/chloroform during homogenation of the fatty brain tissue. Subsequently, the Aurum Total RNA mini Kit (Bio-rad) was used according to the manufacturer’s protocol. RNA dissolved in elution buffer was frozen to -80 °C and shipped to the VIB Nucleomics Core (VIB, Belgium) for RNA sequencing by Next Generation Sequencing technology, i.e. massive parallel sequencing using Illumina Truseq procedures on an Illumina Hi-Seq apparatus.

### Read preprocessing and mapping of RNA-sequencing data

The raw data of FASTQ format were processed to remove as much technical artifacts as possible. Low quality ends (phred score <20) were trimmed using the FASTX tool kit (http://hannonlab.cshl.edu/fastx_toolkit). Adapter trimming was performed with cutadapt 1.2.1 [25]. Reads shorter than 15 bp after adapter trimming were removed. Quality filtering was done using FastX 0.0.13 and ShortRead 1.16.3 [26]. PolyA-reads (more than 90 % of the bases equal A), ambiguous reads (containing N), low quality reads (more than 50 % of the bases < Q25) and artifact reads (all but 3 bases in the read equal one base type) were removed. The remaining clean reads were aligned against the reference genome of *Mus musculus* (mus_musculus_GRCm38.73), using the TopHat v2.0.8b software [27]. As parameter options we used: --library-type fr-unstranded –min-intron-length 50 --max-intron-length 500000 –no-coverage-search --no-mixed --read-realign-edit-dist 3. Quality filtering (removal of reads that are non-primary mappings or have a mapping quality < 20), sorting and indexing of the resulting bam-files were done with samtools 0.1.19 [28].

### Identification of differentially expressed genes

Gene expressions of the transcripts were calculated by counting the number of reads in the alignments that overlap with the gene features, using htseq-count 0.5.4p3 [29]. As parameters we took: -m union --stranded = reverse -a 0 -t exon -i gene id. Subsequently, the edgeR 3.12.0 tool [30] was utilized to detect the differentially expressed genes between the αT-cat WT and KO samples. The modeling of the variance for each gene was done using both the common dispersion model (the same dispersion value is used for each gene) and the moderated tagwise dispersion model (a distinct, individual dispersion is estimated and used for each gene). EdgeR used with the moderated tagwise dispersion model tends to rank more highly as DE genes that are more consistent in their counts within groups. Finally, genes with a *p-value* ≤ 0.01, corresponding to a false discovery rate (FDR) value ≤ 0.3, were considered as differentially expressed (DE).

### Systems biology

The biological interpretation of the DE genes observed in the WT versus αT-cat KO samples was performed using QIAGEN’s Ingenuity^R^ Pathway Analysis (IPA^R^, QIAGEN Redwood City, www.qiagen.com/ingenuity), in order to identify and characterize biological functions, canonical pathways and gene networks associated with these genes. IPA analyses rely on the Ingenuity Pathways Knowledge Base (IPKB), a manually curated database containing data extracted from the full text of journals as well as gene annotation databases and interaction data from third party databases, such as DIP, IntAct, MINT, MIPS, BIND and BIOGRID. An IPA (Core) analysis consists of mapping each gene identifier to its corresponding gene object (focus gene) in the IPKB, the generation of networks using the uploaded focus genes as seeds, identification of the biological processes, diseases, or toxicological functions affected in the experiment (functional analysis) and identification of the canonical pathways the experimental dataset may be involved in (canonical pathways). IPA uses Fisher’s exact test to calculate a *p-*value determining the probability that each biological process, disease or toxicological function assigned to the data set is due to chance alone.

### Statistics

Statistical analysis was performed using Student’s *t*-test, two-tailed and unpaired, with a p-value of less than 0.05 considered significant. Calculations were performed using GraphPad Prism software (Graph Pad Software Inc., La Jolla, CA).

## Results and discussion

### αT-cat is expressed in the cell-cell junctions of ependymal cells, but not the choroid plexus

**Fig. 2:**
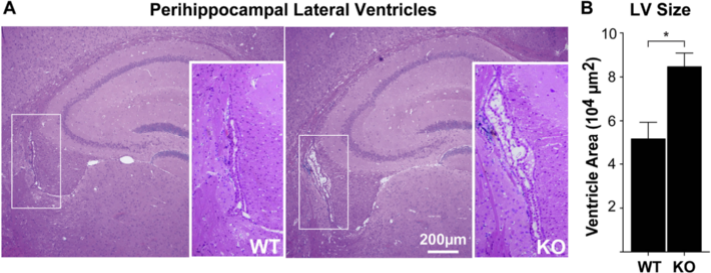
Histologic analysis of lateral ventricle size in WT and αT-cat KO mice. **a** Coronal, H&E-stained sections of WT and αT-cat KO perihippocampal lateral ventricles (enlarged in box inset), with quantification of ventricle size in (**b**), *n* = 4. *p < 0.05, Student’s *t*-test. Error bars = S.E.M

Using littermate matched wild-type (WT) and αT-cat germline knockout (KO) mice, we carried out immunofluorescence analysis of the brain to determine the primary αT-cat expressing cell types. In the cerebrum, we found that αT-cat was predominantly detected in cell-cell junctions of ependymal cells lining the ventricles in mouse (Fig. 1a). Consistent with this observation, we confirmed that αT-cat is also present in the ependyma of human brain (Fig. 1b). Interestingly, αT-cat was not found in the choroid plexus (CP), the only other simple cuboidal epithelium present in brain, which instead expresses αE-cat (Fig. 1a). The localization of αT-cat to cells lining the ventricular-cerebral spinal fluid (CSF) system raised the possibility that αT-cat might impact the structure and function of this system. Loss of αT-cat in ependymal cells was associated with an apparent increase in the abundance of αE-cat (Fig. 1c-d). A similar increase in αE-cat protein is observed in the hearts of αT-cat KO mice [16], suggesting that the upregulation of αE-cat when αT-cat is lost may be a universal compensatory feature of αT-cat loss. While examining the potential changes to ventricle structure, the perihippocampal lateral ventricles appeared mildly enlarged in αT-cat KO mice based on histologic analysis (Fig. 2a-b). Although this suggests that αT-cat may control ventricular size, more robust assays, such as quantitative MRI-imaging, would need to be performed to definitely test whether the loss of αT-cat results in any changes to the ventricular system of the brain.

**Fig. 3:**
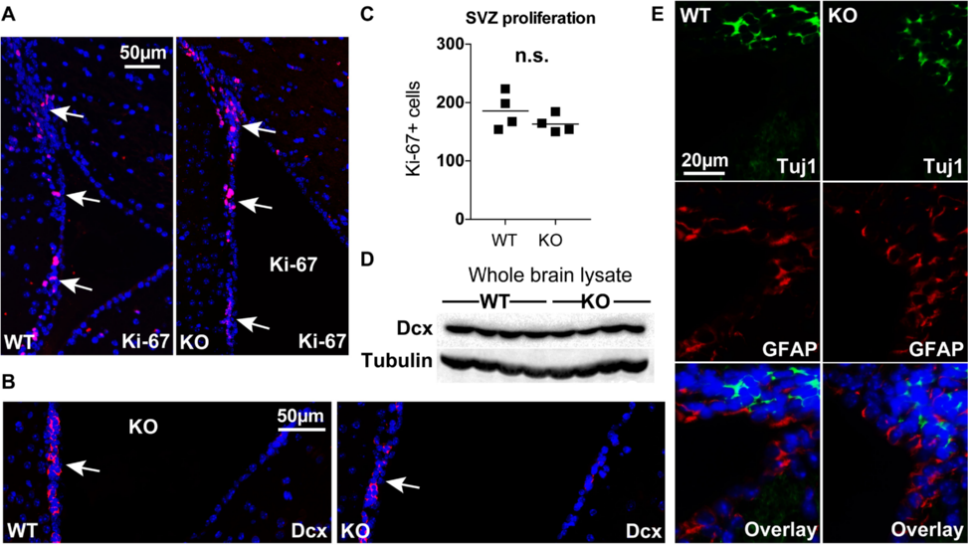
Loss of αT-cat does not disrupt proliferation along the SVZ. **a** Immunofluorescence of the lateral ventricle subventricular zone (SVZ) for Ki-67, indicating similar regions of proliferation (*arrows*) between WT and αT-cat KO mice. **b** Immunofluorescence of doublecortin (Dcx) along the walls (arrows) of lateral ventricle SVZ. **c** Quantification of total Ki-67-positive cells along the WT and αT-cat KO mice SVZ (*n* = 4), with no significant difference by Student’s *t*-test. **d** Western blot of Dcx in whole brain lysate (*below*) of WT and KO mice (*n* = 4), with tubulin as a loading control. **e** Immunofluorescence of focal regions of the lateral ventricle subventricular zone (SVZ) for Tuj1 (green, immature neurons) and GFAP (red, glial cells) of WT and αT-cat KO mice.). n.s. = not significant. Hoechst-stained nuclei in blue

Because the ependymal cells have a well-defined role in regulating neurogenesis [31], we next interrogated differences in the subventricular zone (SVZ) between WT and αT-cat KO mice. However, we found no difference in SVZ proliferation, as measured by Ki-67-positive cells along the walls of the lateral ventricles (Fig. 3a, quantified in Fig. 3c) between WT and αT-cat KO mice. Additionally, when we stained for the neurogenesis marker doublecortin (Dcx), there was no difference observed between WT and αT-cat KO mice by immunofluorescence (Fig. 3b) or by immunoblot analysis (Fig. 3d). Finally, WT and αT-cat KO mice also showed similar glial and immature neuron organization along the lateral ventricles (Fig. 3e). Overall, the loss of αT-cat appears to contribute little effect to the regulation of the SVZ, which is consistent with the lack of gross morphological differences observed in overall brain structure.

### Ependymal cell junctions are morphologically and compositionally distinct from those of the choroid plexus

**Fig. 4:**
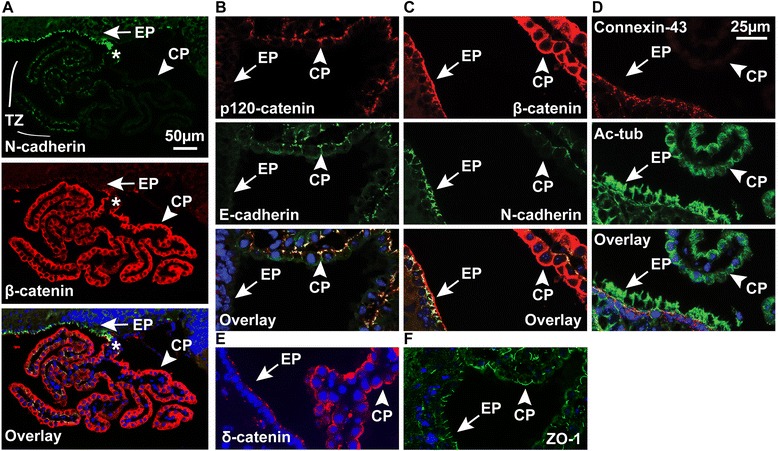
Choroid plexus and ependymal cells have distinct cell-cell adhesion components. **a** Double immunofluorescence of N-cadherin and β-catenin in the choroid plexus and ependyma, showing a cell adhesion transition zone (TZ, starting at the asterisk) **b-f** Double or single immunofluorescence labeling of cell-cell adhesion proteins (p120 catenin and E-cadherin; β-catenin and N-cadherin; connexin-43 and acetylated tubulin, δ-catenin and ZO-1) in ependymal cells (*arrows*) and the choroid plexus (*arrowheads*). Hoechst-stained nuclei in blue

αT-cat expression has only previously been shown in mesenchymal tissues, such as heart [11], and not in traditional epithelia that typically express αE-cat. Therefore, we sought to compare adherens junction protein expression between ependymal cells and choroid plexus (CP), particularly because the latter is long considered to be a modified ependymal cell [32]. Interestingly, we found that the junctions of these epithelia are distinct. When examined by immunofluorescence, there is a transition zone where N-cadherin expression found in ependymal cells decreases along the outgrowth of CP [33], whereas β-catenin expression increases (Fig. 4a). CP was further distinguished by classic epithelial markers, including E-cadherin and p120 catenin (*CTNND1*) (Fig. 4b), while αT-cat-expressing ependymal cell junctions contained N-cadherin and Connexin-43 (Cx-43) (Fig. 4c-d), Interestingly, δ-catenin (*CTNND2*), which has also recently been implicated in autism [34], was detected in both ependymal and CP junctions (Fig. 4e), as was β-catenin (Fig. 4c) and the tight-junction protein ZO-1 (Fig. 4f). Beyond differences in expression of these junction proteins, their subcellular localization was also distinct. While E-cadherin and p120 catenin showed an apicolateral zonular adherens junction localization in the cells of the CP, β-catenin and δ-catenin demonstrated a more basolateral staining. Conversely, N-cadherin and β-catenin displayed an apicolateral junctional staining pattern in the ependyma, while δ-catenin marked the apical membrane.

**Fig. 5:**
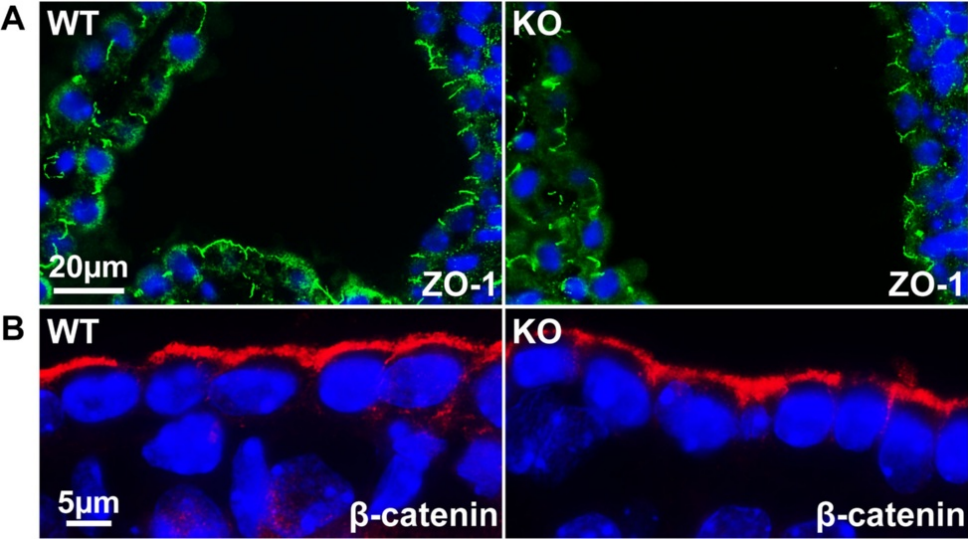
Adherens and tight junctions in the ependyma of WT and αT-cat KO mice. **a** Immunofluorescence of the tight junction protein ZO-1 (*shown in green*) in the ependyma (*shown on right*) and choroid plexus (*shown on left*) of WT and αT-cat KO mice. **b** High-magnification images of immunofluorescence of the adherens junction protein β-catenin (*shown in red*) in the ependyma of WT and αT-cat KO mice. Hoechst-stained nuclei in blue

All together, the ependyma is a morphologically true epithelial layer, but is unique in that it expresses traditional markers of mesenchymal tissue, including N-cadherin and Cx43. Interestingly, these two components also comprise the specialized hybrid adherens junctions αT-cat forms in cardiomyocytes, coordinating the adherens and gap junctions, respectively [16]. Beyond understanding the junctional diversity of the ependyma and CP, these unique expression patterns may also be useful tools in future neurobiology research, as there is currently a dearth of reliable markers to distinguish the CP from the ependyma. Unfortunately, even with all of these cell-cell adhesion proteins found in the ependyma, we could detect no overt differences between these junctional proteins between WT and αT-cat KO mice. The adherens and tight junctions remained intact (Fig. 5), suggesting that the loss of αT-cat may only have subtle effects on ependymal cell-cell junctions.

### αT-cat is detected in the cerebellar, but not the cerebral, cortex

**Fig. 6:**
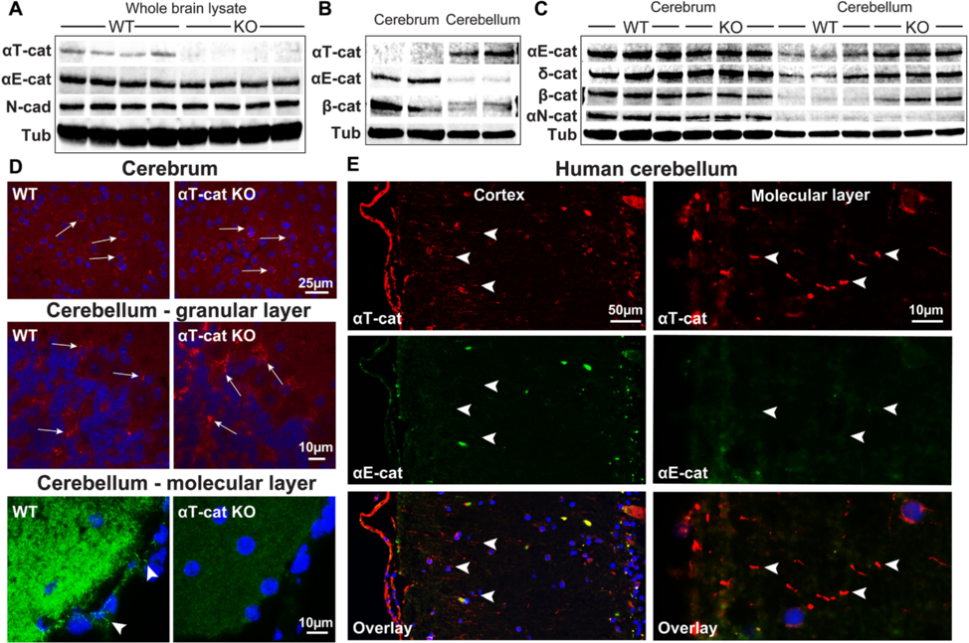
αT-cat is not present in the cerebral cortex, but rather is in the molecular layer of the cerebellum. **a** Western blot of whole brain lysate of WT and αT-cat KO mice. **b-c** Western blot of cerebral cortex and cerebella, dissected without nearby ventricles, of WT and αT-cat KO mice. **d** Immunofluorescence of αT-cat in the cerebral cortex and cerebellum in WT and αT-cat KO mice, with false positive signal (*arrows*) and true positive signal in the molecular layer and pia mater (*arrowheads*) of the cerebellum (*bottom panels*). **e** Double immunofluorescence of αT-cat and αE-cat in human cerebellum, with positive αT-cat staining (arrowheads) in the molecular layer. Hoechst-stained nuclei in blue

We noticed that αT-cat was expressed at relatively low levels by whole brain immunoblot analysis (Fig. 6a), suggesting its presence in rare cells types of the brain. Consistent with αT-cat’s apparently exclusive localization to ependymal cells (Fig. 1), no αT-cat was detected in the cortex of the cerebrum, which was dissected to omit ventricular ependymal contamination (Fig. 6b). αT-cat was also not detected in neurons of the cerebrum, although by immunofluorescence analysis there was a false positive signal in both WT and αT-cat KO mice (Fig. 6d), which may explain previous reports of αT-cat expression in cortical neurons [35]. Interestingly, αT-cat was robustly detected by immunoblot of the cerebellar cortex (also dissected to omit ependymal contamination)(Fig. 6b), suggesting its presence in an abundant cell type. Curiously, while αE-cat expression was dominant in the cerebral cortex, it was much less abundant in the cerebellum (Fig. 6b), suggesting that αT-cat and αE-cat may contribute distinct functions across these brain compartments. Similar to αE-cat, αN-cat was predominantly expressed in the cerebrum and nearly undetectable in the cerebellum (Fig. 6c), which indicates that αT-cat may be the primary α-cat of the cerebellum. Finally, the loss of αT-cat increased the cerebellar expression of αE-cat, β-catenin, and δ-catenin, suggesting a potential compensatory upregulation of these junctional components (Fig. 6c).

Importantly, αT-cat was specifically detected within the molecular- (cellular processes), but not the granular- (cell bodies), layer of the cerebellum, consistent with previous data [36] (Fig. 6d). Using human cerebellar tissue, αT-cat, but not αE-cat, was clearly detected along neurite projections within the molecular layer (Fig. 6e). Although differences in junctional protein expression were found by western blot, overt changes to junctional structures within the cerebellum were absent between WT and αT-cat KO mice (Additional file 1: Figure S1). Since the murine cerebellum did not appear to have the discrete, traditional cell-cell junctions usually visualized by these proteins, the cerebellar function of the altered cell-cell junction proteins shown in Fig. 6c could not be described. While there is some data suggesting that αT-cat and αE-cat may be able to stabilize dendritic spines [37], whether this occurs in the cerebellum is unknown.

Lastly, αT-cat was also found in cell junctions of the pia-arachnoid surrounding the brain (Fig. 6d), which is a simple epithelium that lines the sub-arachnoid space filled with CSF. Since this space is contiguous with the ventricles lined by ependymal cells, these data indicate that αT-cat participates in junctions from simple cuboidal (ependymal) and squamous (pia-arachnoid) epithelia that line this fluid-filled space.

**Fig. 7:**
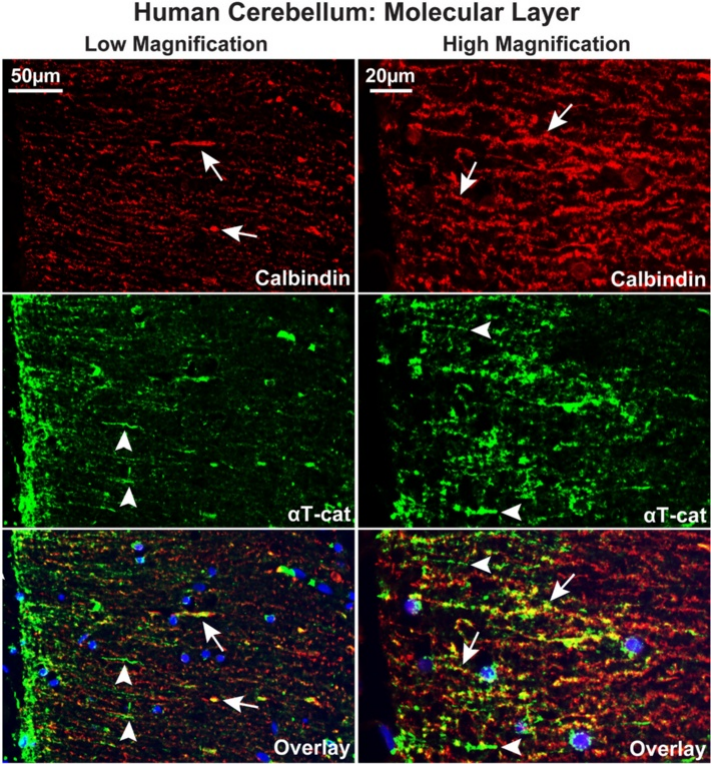
αT-cat is present in diverse cell types in the molecular layer of the cerebellum. Double Immunofluorescence of αT-cat (shown in green) and calbindin D28K (shown in red, marker of Purkinje cells) in human cerebellum, at low (*shown on left*) and high (*shown on right*) magnifications. Areas of both co-localization of αT-cat and calbindin staining (*arrows*) and of αT-cat staining alone (*arrowheads*) can be observed within the molecular layer. Hoechst-stained nuclei in blue

We next sought to identify which cell types in the molecular layer of the cerebellum expressed αT-cat. Neurites from a number of cell types are found in this layer, including the dendritic trees of the Purkinje cells and the projections of granular neurons. Using calbindin D28K as a marker of Purkinje cells [38], immunofluorescence demonstrated that αT-cat in the human cerebellum co-localizes with Purkinje cell dendrites (Fig. 7). Interestingly, αT-cat could also be detected along neurites not calbindin-positive (Fig. 7), indicating that αT-cat may be present in a diverse number of cell types in the cerebellum. Overall, this may be consistent with previous αT-cat promoter-based β-galactosidase expression data [36].

### RNA sequencing analysis of cerebella implicates αT-cat in several neurological disease pathways

While there were no overt morphological or cellular changes found in αT-cat KO mice cerebella when compared to WT, we suspect that the upregulation of cell adhesion proteins in the absence of αT-cat (Fig. 6c) compensates for this loss, as both αE-cat and αT-cat may share similar neural functions [37]. To assess more subtle changes due to the loss of αT-cat that may be relevant to neurological diseases, we performed an RNA-sequencing (RNA-seq) analysis between WT and αT-cat KO mouse cerebella. From this, we discovered a number of gene transcripts altered by the loss of αT-cat, from which we associated with several neurologic disease-relevant pathways. The complete RNA-seq dataset shows these results in complete detail (see Additional file 2). Notable among these were two pathways previously implicated in ASD, including amyloid-β precursor protein APP [39] and estrogen (ESR1) [40] (Additional file 1: Figure S2). Interestingly, the loss αT-cat was associated with changes to a number of other hormone signaling pathways, including corticotropin (CRH) [41] and somatostain (SST) [42], which have also been previously associated with ASD. The cerebellum is not the canonical secretion site for any of these hormones, so αT-cat’s influence on them remains a mystery. One possible hypothesis may be that αT-cat regulates either local production or signaling of these hormones, but further research will need to be conducted to ultimately test this. Finally, notable among the top identified genes influenced by αT-cat was MEIS2 [43], which has also associated with ASD. Importantly, for all of these genes identified by the RNA-seq analysis, each will need to be validated by future experiments to definitively link their expression to αT-cat.

**Fig. 8:**
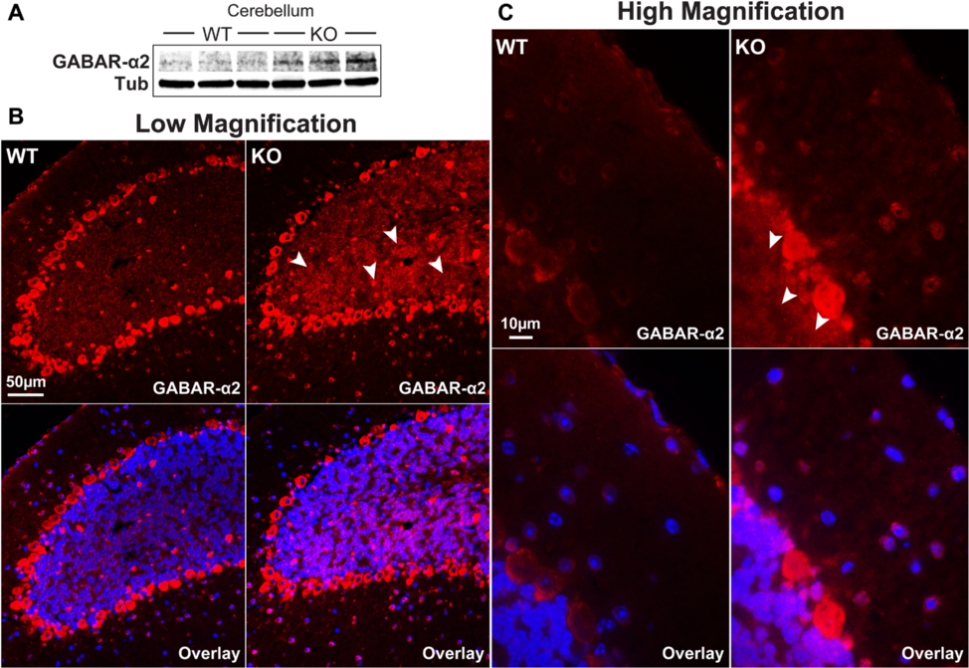
GABA-A receptor α2 expression is increased in the αT-cat KO cerebellum. **a** Immunoblot of WT and αT-cat KO cerebella for the GABA-A receptor α2, with tubulin as a loading control. **b** Immunofluorescence of WT and αT-cat KO mouse cerebella for GABA-A receptor α2 (shown in red), at a low magnification, with a higher magnification shown in (**c**). At both magnifications, more GABA-A receptor α-2 expression can be observed in the granular layer of the cerebellum (*arrowheads*). Hoechst-stained nuclei in blue

Accordingly, we have validated the top associated gene identified by the RNA-sequencing analysis: GABRA2, which encodes GABA-A receptor α2. From the RNA-seq data described above, gene expression of GABRA2 in αT-cat KO cerebella was increased when compared to WT. Consistent with this finding, immunoblot of GABA-A receptor α2 showed increased expression in αT-cat KO mouse cerebella (Fig. 8a). Furthermore, we performed immunofluorescence of WT and αT-cat KO cerebella. Interestingly, the αT-cat KO cerebellum appeared to show an increase in GABA-A receptor α2 expression, primarily within the granular layer (Fig. 8b-c). The role GABA-A receptor α2 may be playing in the granular layer, but how it may directly or indirectly relate to the function of αT-cat, remains unknown. However, previous studies have demonstrated that altered expression of GABA-A receptor α2 has been associated with both autism [44] and schizophrenia [45], suggesting that αT-cat may participate in a shared pathogenesis.

### αT-cat may be linked to neurologic diseases through its ependymal and cerebellar functions

We have shown that αT-cat in the brain is expressed in ependymal cells and the neuronal processes within the molecular layer of the cerebellum. A common feature of these distinct cell types may be their proximity to mechanical stressors, consistent with αT-cat’s function in heart. For example, the ependyma and pia-arachnoid are subject to CSF-flow [46], and the granular neurons of the cerebellum are considered to withstand substantial mechanical forces during development to generate stereotypic cerebellar folds [47]. These region-specific localizations of αT-cat may also have implications for the many genetic linkages between αT-cat and disease. The role of the cerebellum in the development of autism is well defined [48], and our data suggest that αT-cat may play an important role in regulating the cell junction components in neurite structures or cell signaling within the cerebellum. Because we did not detect αT-cat in neurons of the cerebral cortex, and αT-cat KO mice demonstrate no overt neurologic dysfunction, we suspect there is no direct neuronal link of αT-cat dysfunction to the established, neuronal etiologies of ASD, or even AD, such as neurofibrillary tangles or amyloid plaques [49]. However, there is evidence that enlarged ventricles are associated with late-onset AD [50], as well as ASD [51]. Whether this is a secondary effect of neural loss or an independent driver of disease remains unknown, but our evidence that αT-cat comprises the junctions that line ventricles raises the formal possibility that an underlying ependymal barrier defect may contribute to ventricular dysfunction in humans. This may also explain why αT-cat polymorphisms were only associated with sporadic, late-onset AD [17] rather than familial AD. Furthermore, although ASD and AD represent diseases that differ significantly in pathogenesis, our data add to the growing evidence of shared biochemical pathways, particularly in the cerebellum, that link these diseases [52]. Lastly, our finding of αT-cat in ependymal junctions may explain αT-cat’s connection to maternal CMV infection and schizophrenia [20, 21]. Because neonatal CMV primarily infects the ependymal layer [53], and ventricle enlargement has long been associated with schizophrenia [54], αT-cat ependymal junction dysfunction might affect susceptibility to CMV infection and subsequent development of schizophrenia.

## Conclusions

In summary, we show that αT-cat is expressed in ependymal epithelial cells and cellular processes within the molecular layer of the cerebellum, and propose how alteration of these structures may rationalize the various genetic associations between αT-cat and ASD, AD and schizophrenia. Our work also highlights the emerging importance and disease-relevance of this understudied α-cat family member, which has been long considered to be a redundant, non-essential component of adherens junctions.

## Abbreviations

AD, Alzheimer’s disease; ASD, autism spectrum disorder; CMV, cytomegalovirus; CP, choroid plexus; CSF, cerebrospinal fluid; KO, knock-out; WT, wild-type; α-cat, alpha-catenin.

## Additional files

Additional file 1: Figure S1: Localization of β-catenin in the cerebella of WT and αT-cat KO mice. Immunofluorescence of the adherens junction protein β-catenin (shown in green) in the cerebella of WT and αT-cat KO mice. Hoechst-stained nuclei in blue. **Figure S2.** Interaction networks of differentially expressed genes in αT-cat KO vs. WT cerebellum. Two interaction networks were developed from APP and ESR1-relevant genes identified from the RNA-seq analysis. Predicted downregulated transcripts are in teal, upregulated transcripts are in red, and downregulated transcripts in green (color intensity reflects fold change). Blue lines indicate upregulation, gray lines indicate downregulation, and yellow lines indicate inconsistency between findings and database. Full lines show direct interactions, and dashed lines show indirect interactions. (PDF 1561 kb) Additional file 2: Full αT-cat KO vs. WT RNA-sequencing results and analysis (Additional File 1.xls). This file contains the list of genes identified by the RNA-sequencing analysis, along with the analysis of important signaling and disease relevant pathways implicated by those genes identified. (XLS 454 kb)
