# Supplementary material for: αT-catenin in restricted brain cell types and its potential connection to autism
Source: J Mol Psychiatry. 2016 Jun 21;4:2. doi: 10.1186/s40303-016-0017-9 (PMC4915096; doi:10.1186/s40303-016-0017-9)

Folmsbee et al., SUPPLEMENTAL LEGENDS AND FIGURES

Figure S1: Localization of β-catenin in the cerebella of WT and αT-cat KO mice. Immunofluorescence of the adherens junction protein β-catenin (shown in green) in the cerebella of WT and αT-cat KO mice. Hoechst-stained nuclei in blue.

Figure S2: Interaction networks of differentially expressed genes in αT-cat KO vs. WT cerebellum. Two interaction networks were developed from APP and ESR1-relevant genes identified from the RNA-seq analysis. Predicted downregulated transcripts are in teal, upregulated transcripts are in red, and downregulated transcripts in green (color intensity reflects fold change). Blue lines indicate upregulation, gray lines indicate downregulation, and yellow lines indicate inconsistency between findings and database. Full lines show direct interactions, and dashed lines show indirect interactions.

Additional File 1: Full αT-cat KO vs. WT RNA-sequencing results and analysis (Additional File 1.xls). This file contains the list of genes identified by the RNA-sequencing analysis, along with the analysis of important signaling and disease relevant pathways implicated by those genes identified.

Supplemental Figure 1

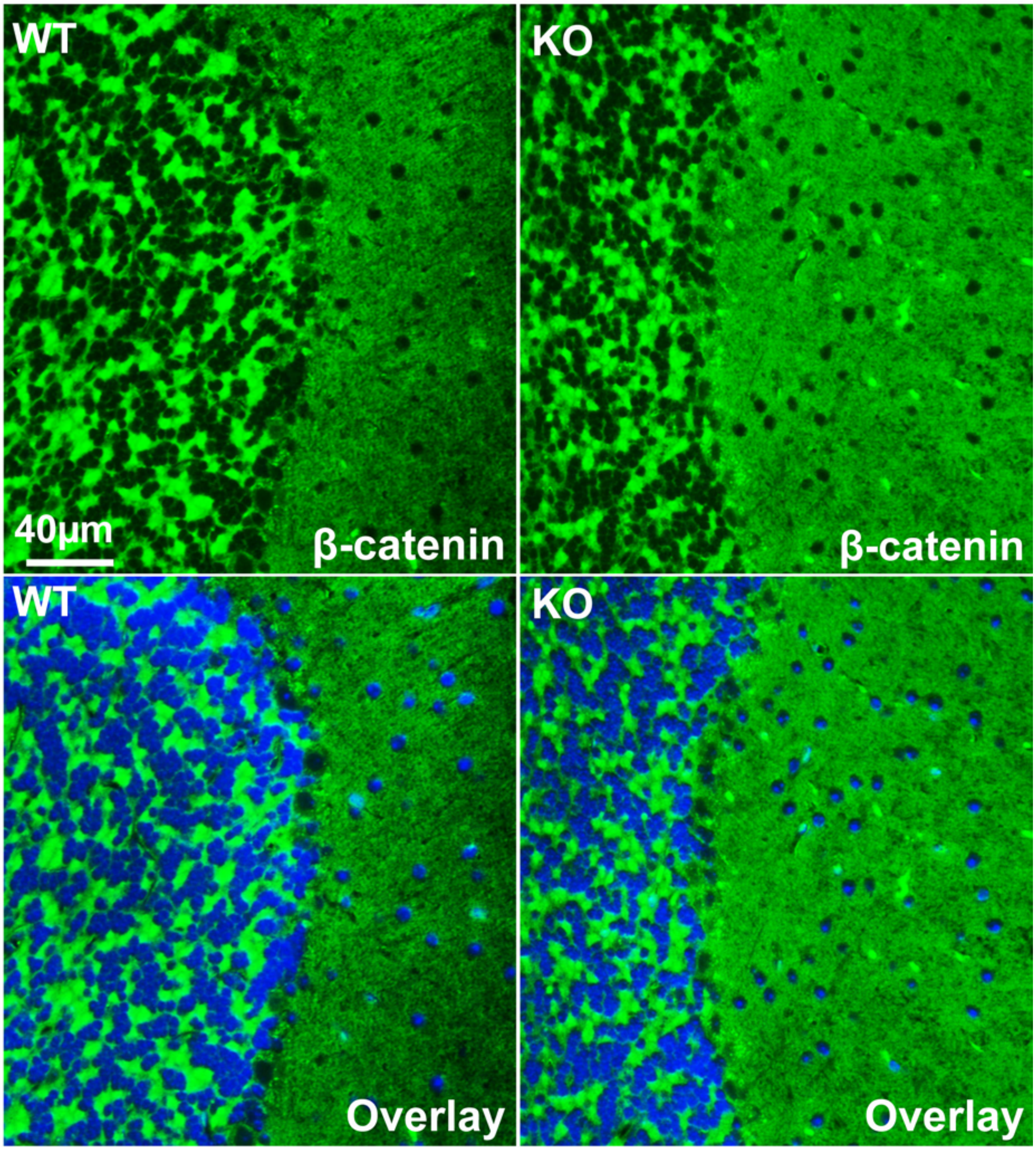

Supplemental Figure 2

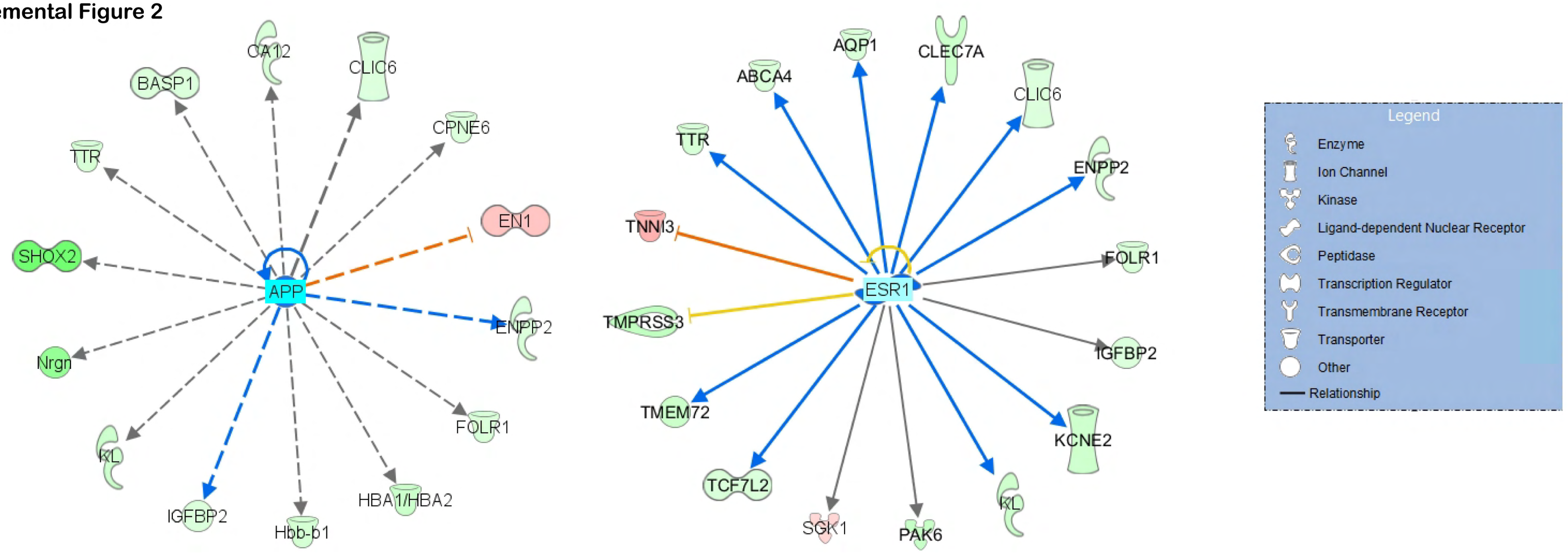

Supplement: Additional file 1: Figure S1: — Localization of β-catenin in the cerebella of WT and αT-cat KO mice. Immunofluorescence of the adherens junction protein β-catenin (shown in green) in the cerebella of WT and αT-cat KO mice. Hoechst-stained nuclei in blue. Figure S2. Interaction networks of differentially expressed genes in αT-cat KO vs. WT cerebellum. Two interaction networks were developed from APP and ESR1-relevant genes identified from the RNA-seq analysis. Predicted downregulated transcripts are in teal, upregulated transcripts are in red, and downregulated transcripts in green (color intensity reflects fold change). Blue lines indicate upregulation, gray lines indicate downregulation, and yellow lines indicate inconsistency between findings and database. Full lines show direct interactions, and dashed lines show indirect interactions. (PDF 1561 kb) [file 40303_2016_17_MOESM1_ESM.pdf]
